# Parental preference for Haemophilus influenzae type b vaccination in Zhejiang Province, China: A discrete choice experiment

**DOI:** 10.3389/fpubh.2022.967693

**Published:** 2022-11-02

**Authors:** Xianglin Wang, Yi Feng, Qian Zhang, Lihong Ye, Man Cao, Ping Liu, Shimeng Liu, Shunping Li, Juan Zhang

**Affiliations:** ^1^School of Population Medicine and Public Health, Chinese Academy of Medical Sciences, Peking Union Medical College, Beijing, China; ^2^School of Health Policy and Management, Peking Union Medical College, Chinese Academy of Medical Sciences, Beijing, China; ^3^Business School, Sichuan University, Chengdu, China; ^4^Department of National Immunization Program, Chinese Center for Disease Control and Prevention, Beijing, China; ^5^Centre for Health Management and Policy Research, School of Public Health, Shandong University, Jinan, China; ^6^School of Public Health, Fudan University, Shanghai, China; ^7^National Health Commission (NHC) Key Laboratory of Health Technology Assessment, Fudan University, Shanghai, China; ^8^School of Health Care Management, Shandong University, Jinan, China; ^9^National Health Commission (NHC) Key Laboratory of Health, Economics and Policy Research (Shandong University), Jinan, China

**Keywords:** Hib vaccination, discrete choice experiment, parental preference, mixed logit model, Chinese parents, immunization policy

## Abstract

**Background:**

China is the only country in the world that has not included the Haemophilus influenzae type b (Hib) vaccine in its National Immunization Program, making it more difficult to eliminate Hib-related diseases through Hib vaccination. It is necessary to study parental preferences for Hib vaccination to optimize vaccine promotion strategies in China.

**Objective:**

This study aimed to investigate Chinese parental preference for five attributes of Hib vaccination, i.e., the place of origin, effectiveness, adverse event, doctors' recommendation, and the price of full vaccination when making a decision to vaccinate their children under 2 years old.

**Methods:**

A cross-sectional survey was conducted in two cities in Zhejiang Province from November to December in 2020 using a discrete choice experiment (DCE). A mixed logit model was used to estimate participating parents' preference for Hib vaccination attributes included in the DCE. Subgroup analysis and probability analysis were also conducted to capture the heterogeneity and trade-off of parental preference for Hib vaccination.

**Results:**

Data from 6,168 observations were included in the analyses. Parents of children are, on average, more likely to voice a positive preference for Hib vaccination. Such attributes of Hib vaccination as effectiveness and doctor's recommendation have a significant positive influence on parents' preference for Hib vaccination, while imported vaccines, adverse events, and the price of full vaccination have a significant negative influence on parents' preference. Parents with different demographic characteristics also existed heterogeneities in preference for Hib vaccination. Parents will make a trade-off on price if the Hib vaccine has a good performance on effectiveness and safety.

**Conclusion:**

The study found that, regardless of the place of origin of the Hib vaccine, parents with children under 2 years old prefer to compromise on price if the vaccine has a better effectiveness and safety profile. A proactive recommendation from doctors would strengthen their willingness for Hib vaccination. These findings help aid the development of communication strategies with parents for Hib vaccination in China.

## Introduction

*Haemophilus influenzae type b* (Hib), a gram-negative cocobacillus bacterium, occurs mainly in children under 5 years of age (especially in children under 2 years of age) and is a common cause of morbidity and mortality in this group of children (1). Globally, ~8 million cases of pneumonia and meningitis and 371,000 deaths are attributed to Hib each year (2), which raises certain challenges for global public health. In China, the pooled carriage of Hib among healthy children in China is 5.87% (3), results from a study of PCR testing of nasopharyngeal secretions (NPS) in Zhejiang Province among children diagnosed with respiratory infections show a positive rate of Hib of 18.49% (4).

Vaccination has long been the most cost-effective means of preventing and controlling infectious diseases (5). Currently, Hib conjugate vaccines have the potential to reduce overall mortality in children by 4% (6). Thus, Hib vaccination is recognized as an effective way to prevent Hib infection, which has been reported regardless of the level of development and economic status of many countries (1, 7). The widespread use of the Hib vaccine worldwide has reduced the number of children who die from Hib infection by over 90% and is expected to eliminate Hib-related diseases (8). Despite the effectiveness of Hib vaccination in preventing Hib, many cases of invasive Hib disease are still reported each year due to unvaccinated, failed vaccinations, etc. Hib vaccine has a low vaccination rate according to available statistics from Immunization Program Information System in China (9), which has been well below the world average (10). As of 2016, the third dose of the Hib vaccine in China has been < 30%, while the global average reached about 70% (9).

China is currently the only country that has not included the Hib vaccine in its National Immunization Program (NIP) and is among the four countries with the highest number of Hib-related deaths worldwide (11). Specifically, vaccines provided to citizens in China are broadly divided into National Immunization Program (NIP) vaccines and Non-Expanded National Immunization Program (non-NIP) vaccines, where NIP refers to vaccines that the government provides free of charge to citizens and that citizens should be vaccinated in accordance with government regulations; NENPI, also known as category II vaccines, refers to other vaccines that are vaccinated by citizens at their own expense and on a voluntary basis. China has now expanded NIP (including one dose of Bacille Calmette-Guerin vaccine, four doses of the oral live attenuated polio vaccine, etc.) and NIP vaccination rates have reached high levels (12). On the contrary, although non-NIP vaccines play an important role as a supplement or limited alternative to NIP in controlling the corresponding infectious diseases and meeting the health needs of different populations, the voluntary and out-of-pocket nature of non-NIP results in a low vaccination rate (13). Chinese children's parents need to pay for Hib vaccination out of their own pocket, and if other factors interfere, which will undoubtedly lead to an insufficient vaccination rate compared with other countries (13, 14). Of all the vaccines in non-NIP, Hib vaccine coverage is relatively low in China compared with other non-NIP vaccines with similar costs, such as the varicella vaccine (15).

The Changchun Changsheng vaccine incident (CCVI, a vaccine safety and quality event that occurred in Changchun, China in 2018) that occurred in 2018 has caused Chinese parents to become more concerned about vaccinations, and more and more parents are proactively searching for information about the non-NIP vaccine online to determine whether to get their children vaccinated (16). Whereas, prior to the incident, parental decisions for uptake non-NIP vaccines were mainly informed by the recommendation of doctors or friends (14). The Chinese government has issued the Vaccine Administration Law following CCVI that requires doctors to communicate adequately with guardians or recipients during the vaccination process (see Article 45 of the Vaccine Administration Law)^1^. Specifically, doctors must communicate more with guardians prior to vaccination so that guardians know more about the vaccine and vaccination and to confirm that their choice to vaccinate their child is well-informed. In the case of non-NIP vaccines, doctors need to introduce more detail about the vaccine and the benefits of vaccination to ensure that the parent's decision to vaccinate is voluntary. These regulations will enhance parents' awareness of vaccines and potentially change their vaccination habits. It is, therefore, necessary to investigate parental preference of Hib vaccination, which can guide health care professionals to start structuring vaccine conversations with parents.

Discrete choice experiment (DCE), a quantitative attribute-based survey method, is widely used in public health to assess community views and preferences and to measure benefits (utility) (17, 18). The result of DCE can assist policymakers in understanding which characteristics or features of public health programs citizens have the highest preferences (17, 18). Existing studies have widely applied DCE to investigate preferences for different vaccines [COVID-19 vaccination (19), human papillomavirus vaccination (20), infant meningococcal vaccination (21), etc.] on numerous characteristics (effectiveness, cost, etc.). Rare studies applied this method to look at the factors influencing parental preference for Hib vaccination.

In this study, we aim to look at factors affecting the preferences of parents with children aged under 2 years old for Hib vaccination in Zhejiang Province, and conduct a systematic analysis of parental preferences through subgroup analysis drawing on the theory of DCE. To the best of our knowledge, this study represents the first DCE work to investigate parental preference for Hib vaccination in China. As mentioned above, the background that the Hib vaccine was not included in the Chinese NIP and the increased awareness of parents about vaccination encourages us to explore parent preferences for Hib vaccination based on the current situation, which serves as a stepping stone for future research in Hib vaccination in China. The second objective of our study is to propose several insights and policy advice in terms of effectiveness, adverse events, doctor's recommendations, and place of origin of Hib vaccines based on the results of all analyses, which can provide a reference for adjusting and optimizing Hib vaccine immunization strategies in China in the future.

## Materials and methods

### Sampling and study population

This study was conducted in two cities of Shaoxing and Wenzhou in Zhejiang, a developed province in the east of China. And then two districts/counties with per capita GDP ranking in the upper and lower quartiles (P_25, P_75) in 2019 and providing Hib vaccines were selected from each city. Four vaccination clinics were then selected by convenient sampling (i.e., two from the rural area and two from the urban area) in each district/county. Priority is given to vaccination clinics that can supply both domestic and imported Hib vaccines. A total of 16 vaccination clinics were invited to participate in the study. The survey was administered online *via* Wenjuanxing (WJX, https://www.wjx.cn/), an online survey company in China between November and December of 2020. Father or mother of children under age 2 (i.e., born between November 1st of 2018 and November 1st of 2020) was recruited in the observation room after routine vaccination with informed consent, and grandparents and other family members of children were excluded. The sample size was determined according to the equation of $N>500\times \frac{c}{\left(t\times a\right)}$ (17), where the largest number of levels *c* among different attributes in this study was 4, and the number of choice sets *t* and the number of alternatives in each set *a* was 8 and 2, respectively. Therefore, the minimum value of *N* could be estimated as (500 × 4)/(8 × 2) = 125. To ensure that a sufficient number of valid questionnaires are collected, we invited 120 parents in each city (i.e., 15 parents in each vaccination clinic) to file the questionnaire. The study was approved by the *Chinese Center for Disease Control and Prevention Institutional Review Board (#201944)*.

### Experiment and questionnaire design

In the discrete choice experiment, participants are asked to complete a series of questions, and each of which corresponds to a hypothetical scenario (22). Each scenario contains 2 or more attributes with different definitions that have different levels. After the participant understands each attribute and its level in a hypothetical scenario, they need to make a choice between 2 or more options. Participants' preferences for the different levels of each attribute and their willingness to make trade-offs between attributes can be analyzed according to their choices across multiple scenarios (18).

Following methodological guidelines of DCE (23), we first identified the important attributes and levels that influence parental preference for Hib vaccination through a literature review related to Hib vaccination. A study in Thailand, one of the last countries to include the Hib vaccine in the NIP, also found that despite the low burden of Hib-related disease in the country, the adverse event and effectiveness of the vaccine still had a significant influence on parental preference for Hib vaccination (24). Furthermore, although Pneumococcal Conjugate Vaccine (PCV), as a non-NIP vaccine in China, has a higher price than the Hib vaccine, vaccination rates in economically developed Shanghai showed that much higher rates for the first dose of the Hib vaccine significantly lower than the PCV vaccine (25). Accordingly, the factors besides price such as parental knowledge about Hib vaccination and whether or not a doctor recommends it, also are major factors underlying the low coverage (26–29). Based on previously published literature regarding DCE studies on vaccination (27–30), we initially identified 17 attributes, which may influence vaccination decisions. In addition, we conducted face-to-face interviews with 17 key stakeholders (i.e., 4 experts from the national, provincial, and local Center for Disease Control and Prevention, 4 vaccination clinic staff, 8 parents of children, and 1 expert from the DCE field) to assess the appropriateness of attributes and its levels, and rank the attributes by the order of importance. Finally, we selected five attributes, i.e., the place of origin, effectiveness, adverse event, the price of full vaccination, and doctor's recommendation. The levels of these five attributes are listed in Table 1.

**Table 1:** Attributes and levels on the discrete choice experiment.

**Attribute**	**Definition**	**Attribute level**
Place of origin	Type of vaccine manufacturer	Domestic product
		Imported product
Effectiveness (%)	The percentage of children that will be protected against a Hib infection when vaccinated	75%
		85%
		95%
Adverse event	The percentage of vaccinated children that will suffer from severe side effects due to Hib	5/1 million doses (low adverse event)
	vaccination	15/1 million doses (moderate adverse event)
		25/1 million doses (high adverse event)
The price of full vaccination	Price per child for full Hib vaccination	200 yuan
		400 yuan
		600 yuan
		800 yuan
Doctor's recommendation	Whether doctors recommend vaccinations for children	Recommendation
		No recommendation

After defining the attributes and attribute levels, the relative importance of these attributes in the view of parents was evaluated by offering two different vaccination choices with different combinations of attribute levels. Among the five determined DCE attributes, two attributes have two levels, two attributes have three levels and one attribute has four levels, thus 144 possible scenarios (2^2^×3^2^×4^1^) and a total of 10,296 possible pair-wise choices ($\frac{144\times 143}{2}$) were generated in a full factorial design. Based on the DCE design package in SAS software, 24 manageable choice sets were obtained using a sequential orthogonal factorization design technique. To minimize the cognitive burden on participants, the 24 choice sets were further divided equally into three blocks, and each block included 9 pair-wise choice sets. In each block, two choice scenarios (pairs 2 and 9) were set to be the same for checking whether the data met internal consistency, i.e., whether the participants made the same answer for the two choice scenarios.

To maximize the information received from the participants, a pair-wise binary two-stage response DCE design was applied in this study following Marshall et al. (31) and Cheng et al. (32). In the first stage, each participant was asked to choose the preferred choice from two alternative vaccination options. Subsequently, in the second stage, each participant was further confirmed whether they would, in reality, vaccinate their child with the Hib vaccine selected in stage 1. Table 2 shows the example of a choice set, and each participant was asked to respond in both stages.

**Table 2:** Attributes and levels on the discrete choice experiment.

**Attribute**	**Hib vaccine A**	**Hib vaccine B**
Place of origin	Imported product	Domestic product
Effectiveness (%)	85%	75%
Adverse events	5/1 million doses (Low)	25/1 million doses (High)
The price of full vaccination	800 yuan	400 yuan
Doctor's recommendation	No recommendation	Recommendation
*First stage:* Which vaccine would you prefer?	□	□
*Second stage:* In reality, would you vaccinate your child with the option you chosed above?	YES NO	

In the final version of the electronic questionnaire, the socio-demographic characteristics of participants and their knowledge about the Hib vaccine were collected in addition to the designed DCE questions, and the questionnaire is included in the Supplementary material.

### Survey and data collection

After completing the questionnaire design, a pilot study was conducted to check the comprehensibility, acceptability, and effectiveness of the electronic questionnaire prior to the formal study, and the existing problems were further addressed in the formal study.

The subjects of this study were only the father or mother of the children aged 0–2 years (born on or after November 1st, 2018), excluding grandparents and other family members, and the whole investigation process was divided into two stages: pre-investigation and formal investigation. To check the comprehensibility, acceptability, and effectiveness of the complete electronic questionnaire, the convenience sampling method was adopted in the pre-investigation stage. A vaccination clinic in Beijing (Jianwailang Home Community Health Service Center, Chaoyang District, Beijing) was selected to carry out the pre-investigation, and the existing problems in the pre-investigation were further modified.

In the process of investigation, for each participant, a professional investigator would give one-on-one guidance to each participant to scan the QR code of the electronic questionnaire and fill in it by using mobile phones or other convenient mobile devices. Specifically, the team of investigators was formed by the vaccination clinic itself, and they need to be uniformly trained to be competent for this investigation. Each investigator works on three tasks: First, the significance of the investigation, the DCE questions, and other questions should be explained in detail to each participant. Secondly, before filling in the questionnaire, the investigator should explain the contents of the informed consent, and inform the participants that the questionnaire will be filled out anonymously, and the relevant information will not involve personal privacy and confirm the participants' willingness to participate in this investigation. For participants who agree to participate in this investigation, they should be asked to fill out the questionnaire truthfully. Finally, the investigator should fill in the vaccination clinic code correctly so that the number of completed questionnaires for each clinic can be checked in real-time in the database, and the questionnaire administration time for each participant should be limited to 20–30 min. After the successful completion of the pre-investigation, a formal investigation was carried out in 16 vaccination clinics in Zhejiang Province.

### Statistical analysis

We used a most promising state-of-the-art discrete choice model, the mixed logit model (20, 30, 33), to estimate parental preferences for the different levels of attributes. It considered repeated choices by the same participant and allowed for random coefficients at the respondent level. Participants' preferences for all levels of each attribute (including the reference group) were estimated using effect coding. Specifically, the mixed logit model is constructed based on a random utility theory framework. The utility for the participant *i* derives from choosing alternative *j* in choice scenario *t* can be calculated as follows:


$$\begin{array}{l} U_{ijt}=X_{ijt}\beta_{i}+\varepsilon_{ijt} \end{array}$$

where *X*_*ijt*_ denotes a vector of observed attributes of alternative *j* in choice scenario *t* (i.e., Hib vaccination preferences attributes and corresponding levels); *β*_*i*_ represents a vector of individual-specific coefficients that reflect the preferability of the attributes; the multiplication of *X*_*ijt*_ and *β*_*i*_ represents the fixed utility of participant *i* choosing alternative *j* in choice scenario *t*; *ε*_*ijt*_ denotes a random utility of participant *i* choosing alternative *j* in choice scenario *t*.

Among the five designed DCE attributes, the price is coded as a continuous variable and the other four attributes are coded as dummy variables. Thus, the utility *U*_*ijt*_ that participant *i* derives from choosing alternative *j* in choice scenario *t* can be calculated as:


$$\begin{array}{l} U_{ijt}=\beta_{0}imported\;product+\beta_{1}effect\;85+\beta_{2}effect\;95\\ \;+\beta_{3}moderate\;adverse\;event+\beta_{4}high\;adverse\;event\\ \;+\beta_{5}^{*}recommendation+\beta_{6}price+\beta_{7}ASC\_None+\varepsilon_{ijt} \end{array}$$

In the equation, *ASC*_*None* is an alternative specific constant for choosing not to vaccinate (i.e., opt-out) (34), and the reference group is set up as follows: imported product, effectiveness with 75%, low adverse event, and no recommendation for the doctor' advice.

We first estimated the main effects mixed logit model to assess parental preferences for the different levels of attributes compared with the reference group. To consider preference heterogeneity, each coefficient was presented as having a mean and a standard deviation, and the mean denotes the overall average preference, and the standard deviation is the individual-specific preference. We also performed subgroup analyses from 4 perspectives to capture differences in Hib vaccination preferences among participants with different characteristics (child residence, parental highest educational attainment, occupation, past history of experiencing adverse events for child). Results from the main effects mixed logit model was also employed to analyze the percentage change in the probability of choosing that specific alternative compared to the base alternative by changing the level of a given attribute. Initial data obtained from the electronic questionnaires were pre-processed using Python software, and all analyses were performed using Stata 15.

## Results

### Study participants

A total of 257 parents of children aged 0–2 years old participated in the study. To ensure internal consistency of the data, i.e., the participants filled in the electronic questionnaire rationally, the results of the choice test for all participants in the duplicated choice tasks were examined, and 38 out of all participants failed in the consistency test. The characteristics of participating parents and their children are shown in Table 3. Based on comparisons with demographic characteristics of participants, there are no statistically significant differences between the participants who failed in the consistency test and those who passed the consistency test. A total of 219 participants who passed the consistency test were included in the data analysis. Most participants were mothers of children, aged between 25 and 34 years, had attained high educational attainment, had earned between 5,000 RMB (yuan) and 9,999 RMB (yuan) monthly, employed by non-healthcare institutions. Fewer children of the participants who were transient population, third child, had experienced adverse events in previous vaccinations.

**Table 3:** Demographic characteristics.

**Demographic information**	**All samples**		**Consistent samples**		**Inconsistent samples**		**Statistical performance**	
	***n*** = **257**		***n*** = **219**		***n*** = **38**			
	***n***	**%**	***n***	**%**	***n***	**%**	**chi-squared**	***p*-value**
**Parent**
**Relationship with child**							0.494	0.482
Father	44	17.1	39	17.8	5	0.1		
Mother	213	82.9	180	82.2	33	0.9		
**Highest education attainment**							3.666	0.453
Junior high school and below	40	15.6	35	16	5	0.1		
High school	60	23.3	55	25.1	5	0.1		
College	74	28.8	60	27.4	14	0.4		
Bachelor	82	31.9	68	31.1	14	0.4		
Master and above	1	0.4	1	0.5	0	0		
**Age, years old**							0.287	0.866
<25	28	10.9	23	10.5	5	0.1		
25–34	184	71.6	157	71.7	27	0.7		
≥35	45	17.5	39	17.8	6	0.2		
**Occupation**							0.721	0.396
Healthcare related profession	36	14	29	13.2	7	0.2		
Non-healthcare related profession	221	86	190	86.8	31	0.8		
**Monthly income/per** ^**a**^							6.893	0.229
<¥2,500	19	7.4	19	8.7	0	0		
¥2,500 – ¥4,999	49	19.1	42	19.2	7	0.2		
¥5,000 – ¥9,999	116	45.1	95	43.4	21	0.6		
¥10,000 – ¥19,999	47	18.3	40	18.3	7	0.2		
¥20,000 – ¥34,999	12	4.7	12	5.5	0	0		
≥¥35,000	14	5.4	11	5	3	0.1		
**Gender**							0.659	0.417
Boy	117	45.5	102	46.6	15	0.4		
Girl	140	54.5	117	53.4	23	0.6		
**Residence**							0.497	0.974
Urban	86	33.5	73	33.3	13	0.3		
Rural	125	48.6	107	48.9	18	0.5		
Mobility	30	11.7	25	11.4	5	0.1		
Not available	11	4.3	10	4.6	1	0		
None	5	1.9	4	1.8	1	0		
**Birth order**							0.756	0.685
First born	144	56	123	56.2	21	0.6		
Second born	109	42.4	92	42	17	0.4		
Third born	4	1.6	4	1.8	0	0		
**Past history of experiencing adverse events following vaccination**							3.097	0.078
Yes	48	18.6	37	16.9	11	28.9		
No	209	81.3	182	83.1	27	71.1		

a ¥1 = $ 0.1565 (November 9, 2021).

### Parental preferences for Hib vaccination

To determine whether there is a difference in the DCE results based on all samples (i.e., consists of consistent samples and inconsistent samples) and consistent samples, we constructed mixed logit models in these two different datasets, respectively. Both models achieve convergence and their results are reported in Table 4, respectively. The main results were similar regardless of the inclusion or exclusion of the participants who did not pass the consistency test. Hence, we only analyze the DCE results based on the consistent samples as follows.

**Table 4:** Mixed logit estimates on Hib vaccination preferences.

**Attribute**	**Consistent samples**				**All samples**			
	***β* ^i^**	**SE**	**95% CI**		***β* ^i^**	**SE**	**95% CI**	
**Mean**
***Place of origin:*** **domestic product (ref.)**
Imported product	−0.189	0.129	−0.442	0.064	−0.122	0.109	−0.335	0.091
***Effectiveness (%):*** **effectiveness 75% (ref.)**
Effectiveness 85%	0.799^***^	0.116	0.573	1.026	0.760^***^	0.104	0.555	0.964
Effectiveness 95%	2.061^***^	0.193	1.683	2.439	1.913^***^	0.166	1.589	2.238
***Adverse events:*** **low adverse event (ref.)**
Moderate adverse event	−0.725^***^	0.129	−0.977	−0.473	−0.659^***^	0.112	−0.879	−0.440
High adverse event	−1.168^***^	0.150	−1.463	−0.874	−1.073^***^	0.129	−1.326	−0.819
***Doctor's recommendation:*** **Not recommended (ref.)**
Recommended	0.468^***^	0.119	0.235	0.701	0.431^***^	0.105	0.226	0.636
***Price***	−0.001^***^	0.000	−0.001	0.000	−0.001^***^	0.000	−0.001	0.000
***ASC (opt-out)***	−9.284^***^	1.369	−11.967	−6.601	−9.560^***^	1.388	−12.280	−6.840
**SD**
***Place of origin:*** **domestic product (ref.)**
Imported product	1.479^***^	0.160	1.165	1.794	1.290^***^	0.131	1.033	1.547
***Effectiveness (%):*** **effectiveness 75% (ref.)**
Effectiveness 85%	−0.035	0.163	−0.355	0.284	0.016	0.155	−0.288	0.321
Effectiveness 95%	1.586^***^	0.185	1.225	1.948	1.511^***^	0.167	1.183	1.839
***Adverse events:*** **low adverse event (ref.)**
Moderate adverse event	−0.013	0.171	−0.348	0.323	0.045	0.175	−0.299	0.389
High adverse event	−0.407	0.270	−0.936	0.121	0.473	0.260	−0.036	0.981
***Doctor's recommendation:*** **no recommendation (ref.)**
Recommendation	1.254^***^	0.143	0.974	1.533	1.176^***^	0.126	0.929	1.423
***Price***	0.002^***^	0.000	0.001	0.003	−0.002^***^	0.000	−0.003	−0.001
***ASC (opt-out)***	5.791^***^	0.739	4.342	7.240	6.527^***^	0.896	4.771	8.282
Samples	219				257			
Observations	5,256				6,168			
Log-likelihood	−1,082.9447				−1,308.6328			

* i: *p*-value < 0.10;

** *p*-value < 0.05;

*** *p*-value < 0.01.

In the discrete choice analysis, the coefficients of four attributes (effectiveness, adverse events, doctor's recommendation, price) at all levels were significantly different from the reference group (*p-*value < 0.05), suggesting these four attributes were meaningful on parental preference for Hib vaccination (Table 4). On the contrary, the coefficient of imported products is not significantly different from domestic products revealing that it was not meaningful to participants on the preference of Hib vaccination.

The relative preferences of participants for different levels of attributes are important in explaining the experimental results of the DCE. The coefficient of effectiveness with different levels showed that the positive influence of effectiveness with 95% is greater than effectiveness with 85% compared with effectiveness with 75%. Similarly, the coefficient of adverse events with different levels showed that the negative influence of the high adverse event on parental preference for Hib vaccination is greater than the moderate adverse event. Parents also had strong preferences for Hib vaccination for doctor's recommendation and Hib vaccines with low price. Out of all five attributes, the adverse events exist homogeneous preferences (*p*-values > 0.05 in the estimated standard deviation of the mean coefficients), and the remaining four attributes all have unobservable preference heterogeneity. When we examined the coefficient of ASC-None, we found a significant negative influence. The result indicated that parents are, on average, more likely to voice a preference to choose Hib vaccination for their children, regardless of the level presented by the other attributes.

### Variation in the parental preference for Hib vaccination

To capture differences in Hib vaccination preferences among participants with different characteristics, we performed subgroup analyses from 3 perspectives (occupation, parental highest educational attainment, past history of experiencing adverse events for child), and all results can be found in the Supplementary Tables A1–A3.

Subgroup analyses revealed several heterogeneities in preferences for Hib vaccination across the following perspectives (in the Supplementary Tables A1–A3). Unlike the overall results, the doctor's recommendation and price do not influence the parental choice for the Hib vaccination for their children who experienced adverse events in the past vaccinations, and only effectiveness and the adverse event can drive their decision. When parents are healthcare-related practitioners, their preference for Hib vaccination is influenced only by effectiveness and adverse events. Parents in other occupations are additionally influenced by price and doctor's recommendation in their preferences for Hib vaccination. Moreover, parents with junior high school education or below preferred domestic products. Among parents with a bachelor and above and children with adverse events in the past vaccinations the results showed that price and doctors' advice were not meaningful to them.

### Probability analyses

**Figure 1:**
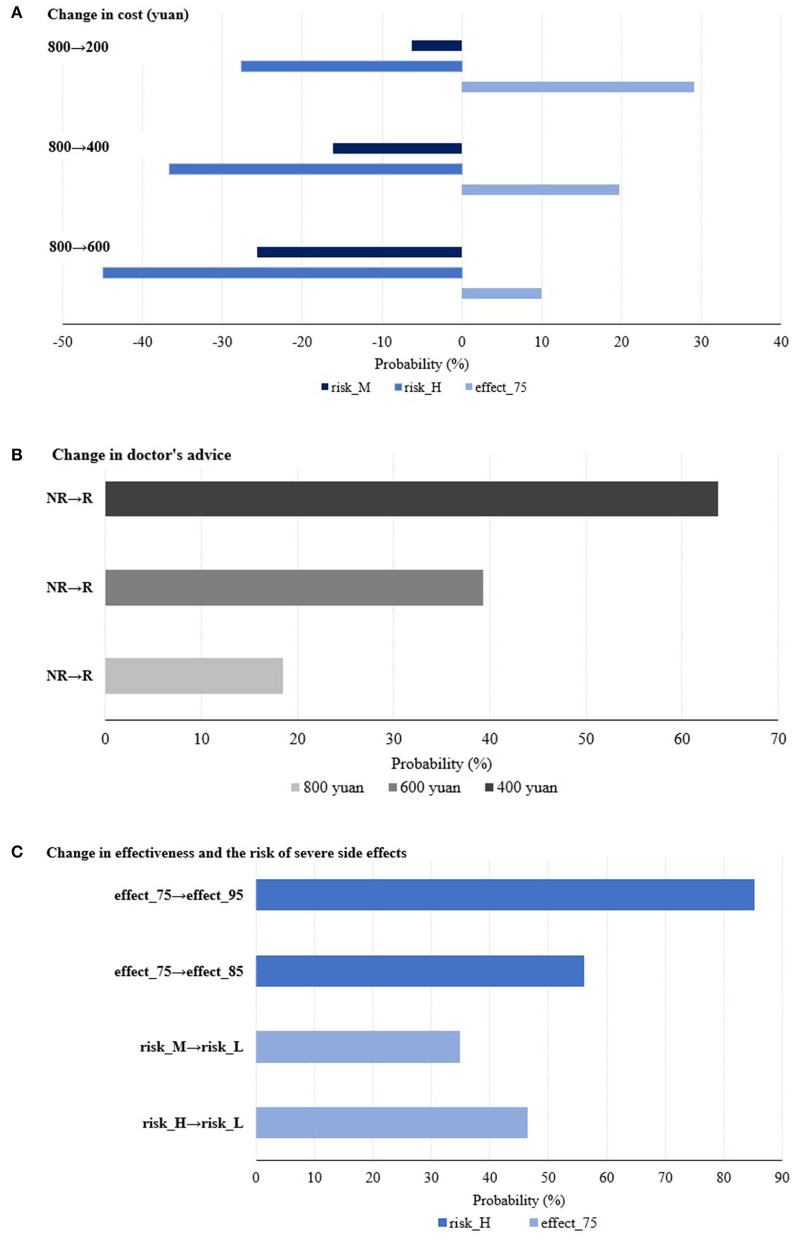
Simulated preferences for Hib vaccination under various potential cases.

Probability analysis, a simulation method, is also utilized to analyze the percentage change in the probability of choosing that specific alternative compared to the base alternative by changing the level of a given attribute. Figure 1 illustrates these changes from three perspectives: changes in price, changes in doctor's recommendation, and changes in effectiveness and adverse events. Taking Figure 1A as an example, the dark blue bars mean that the probability of participants choosing the Hib vaccine with risk_M drops by about 6, 16, and 26% when the cost changes under three different cases (from 800 to 200 yuan; from 800 to 400 yuan; from 800 to 600 yuan), respectively. The other bars can be understood in the same way.

In relation to the levels of price (as shown in Figure 1A), when the price drops by 200 from 800 yuan, parents are 29.13% more likely to be willing to accept a Hib vaccine with 75% effectiveness. When the doctor's recommendation changed from not recommending to recommending for Hib vaccination under different prices (800, 600, and 400 yuan), parents are 18.45, 39.33, and 63.70% more likely to choose Hib vaccination for their children, respectively (as shown in Figure 1B). For the trade-off between effectiveness and the adverse event (as shown in Figure 1C), parents are 85.23% more likely to be willing to trade a low adverse event for a vaccine with a high adverse event when the effectiveness of the vaccine is increased from 75 to 95%, and 46.42% more likely to be willing to trade 95% effectiveness for a vaccine with 75% effectiveness when the adverse event decreases from high to low.

## Discussions

### Principal findings

Among all non-NIP, Hib vaccine coverage is relatively low in China compared with other paid vaccines with similar costs [i.e., overall coverage of 61.1% for the full process varicella vaccination (15)]. In addition to the key factor that the Hib vaccine is part of non-NIP, there are a variety of other factors that come into play, such as parental knowledge, the cost of the vaccine, and whether or not the doctor recommends it. Despite prior studies investigated the four vaccine attributes on people's preference for vaccination (20, 30, 31, 35), the doctors' recommendation was ignored. Due to the crisis of confidence caused by the CCVI event and the low awareness of the Hib vaccine among Chinese health care providers may lead to changes in parental preference for vaccination (36, 37), doctor's recommendation was also considered in our study. Therefore, this study investigated parental preferences for Hib vaccination on four vaccine attributes (origin of vaccines, effectiveness, adverse event and price) and one non-vaccine attribute (doctor's recommendation). The results show that even though the Hib vaccine requires payment, Chinese parental attitudes toward vaccinating their children with the Hib vaccine are still positive. This finding is similar to the result of other scholars on the willingness to vaccinate EV71 vaccines at a similar price (38). In line with the findings of existing studies (31), our study also shows that effectiveness and adverse event all have a significant influence on parental preference for Hib vaccination. However, our study reveals several new findings that doctor's recommendation has a significant positive influence, while imported vaccines do not influence parental preference for Hib vaccination.

### Possible explanations and understandings

Results showed that the origin of the vaccine had no significant influence on parental preference for Hib vaccination, which deviates from our expectations and the findings of existing studies (39, 40). It could be influenced by complex factors such as increased positive media coverage of the vaccine prior to the investigation to counter the negative impact of CCVI. The finding could be a positive sign for the inclusion of the Hib vaccine in the NIP, there is after all only one imported Hib vaccine manufacturer in mainland China at present and it may be difficult to make the vaccination widely available. It is also a reminder that there is no need to deliberately emphasize the origin of the vaccine in the publicity of the vaccine.

We found that parents regard the effectiveness of the Hib vaccine as more important than its adverse event, contrary to the findings of a study by Chinese researchers on flu vaccines (41). Perhaps because their previous vaccination experience makes them unconvinced that the vaccine can have serious adverse events, and their perception of benefit from vaccination is not as clarified as that of medical professionals, they would prefer a Hib vaccine with more pronounced effectiveness. Regardless of parents' occupational and social roles, effectiveness and adverse events remained the two most important factors influencing parents' preference for Hib vaccination. Especially for parents whose occupation is healthcare-related, their preference for Hib vaccination is not influenced by the origin of the vaccine, doctor's recommendation and price, because they are more likely to make a choice based on their own cognitions. Additionally, for the price of Hib vaccination, parents will compromise on price due to effectiveness and safety. Lowering the price of the Hib vaccine would also assist in boosting its uptake, meaning that the NIP inclusion of the Hib vaccine in more economically developed or cost-effective areas is urgently needed.

Due to the crisis of confidence caused by the CCVI event and the low awareness of the Hib vaccine among Chinese health care providers (36, 37), the doctor's recommendation on the parental preference for Hib vaccination was also investigated. Previous non-NIP vaccination successes in China have largely been observed by parents following and trusting doctor's recommendations on vaccination (42, 43), similar findings were found in our study. But interestingly, the subgroup analysis showed that parents with higher socioeconomic levels are not significant to be influenced by doctor's recommendations, possibly because they have higher expectations of doctors' service capacity, suggesting the importance of doctors' improved service capacity. Adults' vaccination decisions are mostly irrational and behavioral interventions have influenced their vaccination preferences (44). So various pre-vaccination services should be used by doctors to recommend Hib vaccination and safety information to parents as in vaccination information packs for parents of newborns who are pregnant, parent education sessions held, etc., could be a very efficient intervention to increase Hib vaccination rates (45).

Factors, such as restrictions on the timing of Hib vaccination, the number of doses required, and the fact that the Department of Health does not assess non-NIP vaccination rates, may have led to the little incentive for doctors to recommend vaccines. Hib-containing combination vaccines or co-administration with other NIP vaccines has promoted the Hib vaccine coverage rate in China to a certain extent (46), which deserves further dissemination to change and optimize the current inflexible immunization strategy in China. Targeted incentives from the health sector are also needed to motivate doctors to proactively recommend Hib vaccination.

### Implication for Chinese doctors and government

At present, Chinese citizens can only pay for the Hib vaccine, but it is very necessary to improve the coverage of the Hib vaccine in China. Therefore, the society and vaccination clinics should strengthen the publicity of the safety and effectiveness of the Hib vaccine and increase the enthusiasm of doctors to actively inform and recommend it (informing about the dangers of the disease caused by Hib and the benefits of Hib vaccination), which is a high guideline to increase the willingness to vaccinate with Hib vaccine (47). For the Chinese government, although the Hib vaccine is not included in NIP vaccines, it does not mean that Hib vaccination is not important and not advocated in China, in recent years the Chinese authorities have been attempting to develop plans to implement Hib vaccination in the NIP (46, 48). Therefore, we also call on government officials to make some changes based on the key findings from this study. First, with reference to foreign practices of periodic monitoring of the safety and quality of vaccines, government officials could adequately disclose the safety data and safety survey results of vaccines to ensure parents' confidence in the quality of domestic vaccines. Second, a relevant co-immunization with other vaccines policy from the authorities as soon as possible is also necessary. It would help avoid parents from missing or forgetting the Hib vaccination schedule due to repeated clinic visits, and it would also reduce to some extent the hindrance and concern of doctors in recommending the Hib vaccine. Finally, the Chinese government has, as a recent reform measure, allowed vaccination clinics to charge a fixed fee for medical services from parents who choose to have their children vaccinated with non-nip vaccines (see Article 49 of the Vaccine Administration Law)^1^. However, the and doctors are allocated only a small percentage of it, which is not conducive to doctors' initiative. Doctors may therefore be more motivated to recommend non-NIP vaccine if the fee allocation percenntage could be increased.

### Limitations

In this study, the influence of parental attitudes and cognitions on Hib vaccination also is investigated through parental evaluation of individual attributes. We also effectively observe and analyze the trade-offs and decisions made by parents in Hib vaccine attribute changes using DCE according to their repeated choice results in various hypothetical scenarios. It is no denying that this study exists some limitations. Different from other similar studies (31, 49), we did not examine the parental willingness to pay for Hib vaccination. Because the current cost level of Hib vaccines produced by different manufacturers is 80–200 yuan/dose, which is at a relatively low level among the more popular paid vaccines. Thus, a study of willingness to pay on this basis can lead to some large errors. Additionally, due to the relatively low coverage rate of Hib vaccination in China, this study only surveyed the two cities with higher levels of disposable income in China (Wenzhou and Shaoxing). Admittedly, this work helps us to get positive results more efficiently, but the findings may be limited in extrapolation.

## Conclusions

Effectiveness, adverse events, price and doctor's recommendation are significant attributes when parents are making the decision of whether to choose Hib vaccination for their child. Chinese parental preference for Hib vaccination that emphasizes parents are more willing to compromise on price if the vaccine is more effective and safer. And doctors' proactive recommendations for Hib vaccination enhance parental perceptions of the importance of the Hib vaccine. Regardless of the place of origin of Hib vaccines, parents prefer to choose Hib vaccines that have better effectiveness and safety profile and are cheaper for their children. Furthermore, subgroup analysis reveals that parents with different demographic characteristics existed heterogeneities in preference for Hib vaccination. These significant findings will contribute to the development and optimization of future immunization strategies for Hib vaccination in the future in China.

## Data availability statement

The raw data supporting the conclusions of this article will be made available by the authors, without undue reservation.

## Ethics statement

The studies involving human participants were reviewed and approved by Chinese Center for Disease Control and Prevention Institutional Review Board (#201944). The patients/participants provided their written informed consent to participate in this study.

## Author contributions

XW and YF contributed equally to the problem of the study, preprocessed data, performed the experiment, significantly to analysis and manuscript preparation, performed the data analyses, and wrote the manuscript. QZ, LY, and MC prepared and wrote ethics application materials, designed the discrete choice experiment questionnaire, and collected data. PL and SLiu helped design the discrete choice experiment questionnaire and helped perform the analysis with constructive discussions. SLi contributed to the problem of the study. ZY and JZ contributed to the problem of the study and revised the manuscript, and provided financial support. All authors contributed to the article and approved the submitted version.

## Funding

This research was funded by the China Medical Board (CMB), grant number 20-379. JZ was funded by a Disciplines Construction Project: Multimorbidity (WH10022022034).

## Conflict of interest

The authors declare that the research was conducted in the absence of any commercial or financial relationships that could be construed as a potential conflict of interest.

## Publisher's note

All claims expressed in this article are solely those of the authors and do not necessarily represent those of their affiliated organizations, or those of the publisher, the editors and the reviewers. Any product that may be evaluated in this article, or claim that may be made by its manufacturer, is not guaranteed or endorsed by the publisher.

## Footnotes

^1^The National People's Congress of the People's Republic of China. Vaccine Administration Law of the People's Republic of China. 2019. Available online at: http://www.npc.gov.cn/npc/c30834/201907/11447c85e05840b9b12c62b5b645fe9d.shtml (accessed 30 September 2022).
