# Supplementary material for: Parental preference for Haemophilus influenzae type b vaccination in Zhejiang Province, China: A discrete choice experiment
Source: Front Public Health. 2022 Nov 2;10:967693. doi: 10.3389/fpubh.2022.967693 (PMC9710483; doi:10.3389/fpubh.2022.967693)
Supplement: Supplementary file 1 [file Data_Sheet_1.docx]

**SUPPLEMENTARY MATERIAL**

Table A1. Subgroup analyses: Occupation.

|  | Medical & health related practitioners | | | |  | Non-medical & health related practitioners | | | |
| --- | --- | --- | --- | --- | --- | --- | --- | --- | --- |
| Attribute | β | SE | 95% CI | |  | β | SE | 95% CI | |
| **Mean** |  |  |  |  |  |  |  |  |  |
| ***Place of origin:* domestic product (ref.)** | | | | | | | | | |
| imported product | -0.172 | 0.494 | -1.139 | 0.796 |  | -0.209 | 0.136 | -0.475 | 0.057 |
| ***Effectiveness (%):* effectiveness 75% (ref.)** | | | | | | | | | |
| effectiveness 85% | 0.956^**^ | 0.485 | 0.005 | 1.907 |  | 0.804^***^ | 0.122 | 0.565 | 1.044 |
| effectiveness 95% | 2.429^***^ | 0.865 | 0.735 | 4.124 |  | 2.002^***^ | 0.207 | 1.597 | 2.407 |
| ***Adverse events:* low adverse event (ref.)** | | | | | | | | | |
| moderate adverse event | -2.012^***^ | 0.640 | -3.267 | -0.757 |  | -0.590^***^ | 0.134 | -0.852 | -0.328 |
| high adverse event | -2.027^***^ | 0.750 | -3.498 | -0.557 |  | -1.071^***^ | 0.158 | -1.380 | -0.761 |
| ***Doctor's recommendation:* no recommendation (ref.)** | | | | | | | | | |
| recommendation | 0.662 | 0.596 | -0.505 | 1.830 |  | 0.501^***^ | 0.126 | 0.254 | 0.747 |
| ***Price*** | -0.001 | 0.001 | -0.003 | 0.001 |  | -0.001^***^ | 0.000 | -0.001 | 0.000 |
| ***ASC (opt-out)*** | -5.721 | 2.014 | -9.668 | -1.774 |  | -10.036^***^ | 1.556 | -13.085 | -6.987 |
| **SD** |  |  |  |  |  |  |  |  |  |
| ***Place of origin:* domestic product (ref.)** | | | | | | | | | |
| Imported product | 2.116^***^ | 0.739 | 0.668 | 3.564 |  | 1.411^***^ | 0.164 | 1.091 | 1.732 |
| ***Effectiveness (%):* effectiveness 75% (ref.)** | | | | | | | | | |
| effectiveness 85% | 0.127 | 1.109 | -2.047 | 2.301 |  | -0.038 | 0.184 | -0.397 | 0.322 |
| effectiveness 95% | 2.452^***^ | 0.904 | 0.681 | 4.224 |  | 1.595^***^ | 0.232 | 1.140 | 2.049 |
| ***Adverse events:* low adverse event (ref.)** | | | | | | | | | |
| moderate adverse event | -0.635 | 0.648 | -1.905 | 0.635 |  | 0.048 | 0.168 | -0.283 | 0.378 |
| high adverse event | 1.454^*^ | 0.765 | -0.045 | 2.954 |  | 0.458^**^ | 0.264 | -0.059 | 0.976 |
| ***Doctor's recommendation:* no recommendation (ref.)** | | | | | | | | | |
| recommendation | 2.441^***^ | 0.863 | 0.749 | 4.133 |  | 1.202^***^ | 0.151 | 0.907 | 1.497 |
| ***Price*** | -0.002 | 0.001 | -0.005 | 0.000 |  | -0.002^***^ | 0.000 | -0.002 | -0.001 |
| ***ASC (opt-out)*** | 6.208^***^ | 2.292 | 1.715 | 10.701 |  | 6.187^***^ | 0.953 | 4.318 | 8.055 |
| Samples | 29 | | | |  | 190 | | | |
| Observations | 696 | | | |  | 4560 | | | |
| Log likelihood | -155.95687 | | | |  | -922.07441 | | | |

Table A2: Subgroup analyses: Highest education attainment. ()

|  | Junior high school or below | | | |  | High school | | | |  | College | | | |  | Bachelor or above | | | | |
| --- | --- | --- | --- | --- | --- | --- | --- | --- | --- | --- | --- | --- | --- | --- | --- | --- | --- | --- | --- | --- |
| Attribute | β | SE | 95% CI | |  | β | SE | 95% CI | |  | β | SE | 95% CI | |  | β | SE | 95% CI | |  |
| **Mean** |  |  |  |  |  |  |  |  |  |  |  |  |  |  |  |  |  |  |  |  |
| ***Place of origin:* domestic product (ref.)** | | | | | | | | | | | | | | | | | | | | |
| imported product | -1.817^***^ | 0.613 | -3.018 | -0.615 |  | -0.427 | 0.282 | -0.981 | 0.127 |  | 0.052 | 0.213 | -0.365 | 0.469 |  | 0.123 | 0.214 | -0.298 | 0.543 |  |
| ***Effectiveness (%):* effectiveness 75% (ref.)** | | | | | | | | | | | | | | | | | | | | |
| effectiveness 85% | 0.101 | 0.361 | -0.606 | 0.808 |  | 0.845^***^ | 0.224 | 0.406 | 1.284 |  | 0.902^***^ | 0.208 | 0.494 | 1.310 |  | 1.017^***^ | 0.255 | 0.517 | 1.517 |  |
| effectiveness 95% | 0.838 | 0.562 | -0.262 | 1.939 |  | 2.251^***^ | 0.407 | 1.454 | 3.049 |  | 2.070^***^ | 0.353 | 1.378 | 2.761 |  | 2.766^***^ | 0.440 | 1.903 | 3.629 |  |
| ***Adverse events:* low adverse event (ref.)** | | | | | | | | | | | | | | | | | | | | |
| moderate adverse event | -0.298 | 0.403 | -1.088 | 0.492 |  | -0.453^*^ | 0.262 | -0.967 | 0.061 |  | -0.667^***^ | 0.233 | -1.123 | -0.211 |  | -1.180^***^ | 0.271 | -1.711 | -0.648 |  |
| high adverse event | -1.016^**^ | 0.475 | -1.948 | -0.085 |  | -0.576^**^ | 0.284 | -1.133 | -0.019 |  | -1.267^***^ | 0.275 | -1.807 | -0.728 |  | -1.640^***^ | 0.341 | -2.308 | -0.971 |  |
| ***Doctor's recommendation:* no recommendation (ref.)** | | | | | | | | | | | | | | | | | | | | |
| recommendation | 0.661 | 0.514 | -0.347 | 1.669 |  | 0.533^**^ | 0.221 | 0.100 | 0.966 |  | 0.586^***^ | 0.214 | 0.167 | 1.006 |  | 0.318 | 0.247 | -0.166 | 0.801 |  |
| ***Price*** | -0.001 | 0.001 | -0.003 | 0.001 |  | -0.002^**^ | 0.001 | -0.003 | 0.000 |  | 0.000 | 0.000 | -0.001 | 0.001 |  | -0.001^*^ | 0.001 | -0.002 | 0.000 |  |
| ***ASC (opt-out)*** | -10.605^***^ | 2.824 | -16.140 | -5.069 |  | -6.348^***^ | 1.431 | -9.153 | -3.544 |  | -7.418^***^ | 2.375 | -12.073 | -2.763 |  | -7.955^***^ | 2.050 | -11.972 | -3.938 |  |
| **SD** |  |  |  |  |  |  |  |  |  |  |  |  |  |  |  |  |  |  |  |  |
| ***Place of origin:* domestic product (ref.)** | | | | | | | | | | | | | | | | | | | | |
| Imported product | 2.360^***^ | 0.687 | 1.014 | 3.706 |  | 1.721^***^ | 0.337 | 1.062 | 2.381 |  | 1.219^***^ | 0.259 | 0.711 | 1.727 |  | 1.156^***^ | 0.303 | 0.562 | 1.751 |  |
| ***Effectiveness (%):* effectiveness 75% (ref.)** | | | | | | | | | | | | | | | | | | | | |
| effectiveness 85% | 0.070 | 0.593 | -1.092 | 1.232 |  | 0.002 | 0.357 | -0.699 | 0.702 |  | 0.040 | 0.326 | -0.598 | 0.678 |  | -0.016 | 0.307 | -0.617 | 0.586 |  |
| effectiveness 95% | -1.891^***^ | 0.671 | -3.205 | -0.576 |  | 1.520^***^ | 0.363 | 0.809 | 2.231 |  | 1.604^***^ | 0.383 | 0.853 | 2.355 |  | -1.689^***^ | 0.353 | -2.380 | -0.998 |  |
| ***Adverse events:* low adverse event (ref.)** | | | | | | | | | | | | | | | | | | | | |
| moderate adverse event | -0.070 | 0.385 | -0.825 | 0.685 |  | 0.056 | 0.263 | -0.459 | 0.571 |  | -0.094 | 0.375 | -0.828 | 0.641 |  | -0.061 | 0.380 | -0.805 | 0.683 |  |
| high adverse event | -0.430 | 0.748 | -1.895 | 1.036 |  | 0.124 | 0.356 | -0.574 | 0.822 |  | -0.296 | 0.502 | -1.279 | 0.687 |  | 1.226^***^ | 0.387 | 0.467 | 1.985 |  |
| ***Doctor's recommendation:* no recommendation (ref.)** | | | | | | | | | | | | | | | | | | | | |
| recommendation | 2.549^***^ | 0.842 | 0.897 | 4.200 |  | 1.130^***^ | 0.289 | 0.563 | 1.697 |  | 1.177^***^ | 0.260 | 0.668 | 1.686 |  | -1.399^***^ | 0.308 | -2.004 | -0.795 |  |
| ***Price*** | -0.004^***^ | 0.001 | -0.006 | -0.001 |  | -0.002^***^ | 0.001 | -0.004 | -0.001 |  | -0.001 | 0.001 | -0.003 | 0.002 |  | -0.002^**^ | 0.001 | -0.004 | 0.000 |  |
| ***ASC (opt-out)*** | 9.590^***^ | 3.359 | 3.006 | 16.174 |  | 3.610^***^ | 0.735 | 2.169 | 5.050 |  | -2.530^***^ | 0.760 | -4.021 | -1.040 |  | -5.848^***^ | 1.451 | -8.691 | -3.004 |  |
| Samples | 34 | | | |  | 56 | | | |  | 60 | | | |  | 69 | | | | |
| Observations | 816 | | | |  | 1344 | | | |  | 1440 | | | |  | 1656 | | | | |
| Log likelihood | -160.468 | | | |  | -276.586 | | | |  | -270.478 | | | |  | -337.055 | | | | |

Table A3. Subgroup analyses: Past history of experiencing adverse events following vaccination.

|  | Experienced adverse events in the past | | | | | No adverse events in the past | | | |
| --- | --- | --- | --- | --- | --- | --- | --- | --- | --- |
| Attribute | β | SE | 95% CI | |  | β | SE | 95% CI | |
| **Mean** |  |  |  |  |  |  |  |  |  |
| ***Place of origin:* domestic product (ref.)** | | |  |  |  |  |  |  |  |
| Imported product | -0.025 | 0.313 | -0.639 | 0.589 |  | -0.216 | 0.137 | -0.485 | 0.052 |
| ***Effectiveness (%):* effectiveness 75% (ref.)** | | |  |  |  |  |  |  |  |
| effectiveness 85% | 0.940^***^ | 0.282 | 0.387 | 1.494 |  | 0.749^***^ | 0.126 | 0.503 | 0.996 |
| effectiveness 95% | 2.568^***^ | 0.531 | 1.527 | 3.609 |  | 1.887^***^ | 0.204 | 1.487 | 2.287 |
| ***Adverse events:* low adverse event (ref.)** | | | |  |  |  |  |  |  |
| moderate adverse event | -0.921^***^ | 0.343 | -1.593 | -0.249 |  | -0.635^***^ | 0.137 | -0.904 | -0.367 |
| high adverse event | -1.175^***^ | 0.368 | -1.896 | -0.454 |  | -1.084^***^ | 0.163 | -1.402 | -0.765 |
| ***Doctor's recommendation:* no recommendation (ref.)** | | |  |  |  |  |  |  |  |
| recommendation | 0.338 | 0.261 | -0.174 | 0.851 |  | 0.491^***^ | 0.132 | 0.233 | 0.749 |
| ***Price*** | -0.001 | 0.001 | -0.002 | 0.001 |  | -0.001^**^ | 0.000 | -0.001 | 0.000 |
| ***ASC (opt-out)*** | -7.707^***^ | 2.908 | -13.406 | -2.008 |  | -9.228^***^ | 2.036 | -13.218 | -5.237 |
| **SD** |  |  |  |  |  |  |  |  |  |
| ***Place of origin:* domestic product (ref.)** | | |  |  |  |  |  |  |  |
| Imported product | 1.200^***^ | 0.322 | 0.569 | 1.831 |  | -1.366^***^ | 0.166 | -1.691 | -1.041 |
| ***Effectiveness (%):* effectiveness 75% (ref.)** | | |  |  |  |  |  |  |  |
| effectiveness 85% | 0.090 | 0.362 | -0.619 | 0.798 |  | -0.006 | 0.192 | -0.383 | 0.371 |
| effectiveness 95% | 1.500^***^ | 0.471 | 0.577 | 2.423 |  | 1.570^***^ | 0.219 | 1.141 | 1.999 |
| ***Adverse events:* low adverse event (ref.)** | | | |  |  |  |  |  |  |
| moderate adverse event | 0.324 | 0.421 | -0.502 | 1.150 |  | -0.064 | 0.219 | -0.436 | 0.309 |
| high adverse event | 0.080 | 0.468 | -0.837 | 0.997 |  | 0.537^*^ | 0.219 | 0.000 | 1.075 |
| ***Doctor's recommendation:* no recommendation (ref.)** | | |  |  |  |  |  |  |  |
| recommendation | 0.988^***^ | 0.369 | 0.264 | 1.712 |  | 1.260^***^ | 0.219 | 0.920 | 1.600 |
| ***Price*** | 0.002^**^ | 0.001 | 0.000 | 0.004 |  | -0.001^**^ | 0.219 | -0.003 | 0.000 |
| ***ASC (opt-out)*** | 4.941^***^ | 1.362 | 2.271 | 7.611 |  | 5.440^***^ | 0.219 | 2.845 | 8.034 |
| Samples | 38 | | | |  | 181 | | | |
| Observations | 912 | | | |  | 4344 | | | |
| Log likelihood | -197.62631 | | | |  | -887.32553 | | | |

**QUESTIONAIRE**

*Participants and their children’s socio-demographic characteristics*

1. **Sex of your child:**

A: Male

B: Female

1. **Your role:**

A: Father

B: Mother

1. **Type of Residence registration of your child:**

A: Urban

B: Rural

C: Mobility (children who are not local hukou but have lived locally for more than 6 months)

D: Children who have lived locally for less than 6 months do not have a registration

E: None

1. **What's the rank of this child in your family:**

A: First child

B: Second child

C: Third child and above

1. **Has your child ever had adverse events after vaccination (including rash, high fever, convulsions, anaphylactoid purpura, etc.):**

A: Yes

B: No

1. **Your highest education:**

A: Junior high school and below

B: High school (Senior high school / technical secondary school / vocational high school)

C: College

D: Bachelor

E: Master or above

1. **Your age:**

A: < 25 years old

B: 25-34 years old

C: ≥ 35 years old

1. **Your occupation:**

A: Medical and health related practitioners

B: Non-medical and health related practitioners

1. **The monthly income of your family:**

A: < 2500 yuan

B: 2500-4999 yuan

C: 5000-9999 yuan

D: 10000-19999 yuan

E: 20000-34,999 yuan

F: ≥ 35, 000 yuan

1. **How many people in your family live together: _______**

**Please read the following material:**

Hib (*Haemophilus influenzae type b*) is a pathogen that only infects humans and is transmitted from person to person through droplets from sneezes/coughs. Hib can cause meningitis, pneumonia, septicaemia, epiglottitis and many other serious illnesses, mainly in children under 5 years of age, especially children under 2 years of age. Hib vaccination is the most effective way to prevent Hib infection. In China, Hib vaccine is currently a non-immunized program vaccine, which is administered to children by parents voluntarily and at their own expense.

**You need to consider the following factors in your answer:**

1) Place of origin: domestic or imported.

2) Effectiveness: which refers to the probability that a vaccinated person will be protected from infection.

3)Adverse events: which refers to the incidence frequency of severe side effects after Hib vaccination.

4) The out-of-pocket cost of full vaccination.

5) Doctor’s advice: whether the doctor in the vaccination clinic recommends Hib vaccination.

Introduction: assuming that the vaccination clinic only provides "Hib Vaccine A" and "Hib Vaccine B" to prevent Hib infection, without considering other factors, please carefully compare the difference between Hib vaccine A and B, and make your choice.

***Version 1:*** *The questionnaire of discrete choice experiments*

Subject 1

| Attribute | Hib vaccine A | Hib vaccine B |
| --- | --- | --- |
| Place of origin | Imported product product | Domestic product |
| Effectiveness (%) | 85% | 75% |
| Adverse events | 5.0/1 million doses (Low) | 25.0/1 million doses (High) |
| The price of full vaccination | 800 yuan | 400 yuan |
| Doctor’s recommendation | No recommendation | Recommendation |
| **Which vaccine would you prefer?** | | |
| **In reality, would you vaccinate your child with the** **option you chosed above?** | YES  NO | |

Subject 2

| Attribute | Hib vaccine A | Hib vaccine B |
| --- | --- | --- |
| Place of origin | Imported product | Domestic product |
| Effectiveness (%) | 75% | 95% |
| Adverse events | 15.0/1 million doses (Moderate) | 5.0/1 million doses (Low) |
| The price of full vaccination | 200 yuan | 800 yuan |
| Doctor’s recommendation | No recommendation | Recommendation |
| **Which vaccine would you prefer?** | | |
| **In reality, would you vaccinate your child with the** **option you chosed above?** | YES  NO | |

Subject 3

| Attribute | Hib vaccine A | Hib vaccine B |
| --- | --- | --- |
| Place of origin | Domestic product | Imported product |
| Effectiveness (%) | 75% | 85% |
| Adverse events | 25.0/1 million doses (High) | 15.0/1 million doses (Moderate) |
| The price of full vaccination | 600 yuan | 200 yuan |
| Doctor’s recommendation | No recommendation | Recommendation |
| **Which vaccine would you prefer?** | | |
| **In reality, would you vaccinate your child with the** **option you chosed above?** | YES  NO | |

Subject 4

| Attribute | Hib vaccine A | Hib vaccine B |
| --- | --- | --- |
| Place of origin | Domestic product | Imported product |
| Effectiveness (%) | 85% | 95% |
| Adverse events | 15.0/1 million doses (Moderate) | 5.0/1 million doses (Low) |
| The price of full vaccination | 400 yuan | 200 yuan |
| Doctor’s recommendation | No recommendation | Recommendation |
| **Which vaccine would you prefer?** | | |
| **In reality, would you vaccinate your child with the** **option you chosed above?** | YES  NO | |

Subject 5

| Attribute | Hib vaccine A | Hib vaccine B |
| --- | --- | --- |
| Place of origin | Imported product | Domestic product |
| Effectiveness (%) | 95% | 75% |
| Adverse events | 15.0/1 million doses (Moderate) | 25.0/1 million doses (High) |
| The price of full vaccination | 600 yuan | 800 yuan |
| Doctor’s recommendation | Recommendation | No recommendation |
| **Which vaccine would you prefer?** | | |
| **In reality, would you vaccinate your child with the** **option you chosed above?** | YES  NO | |

Subject 6

| Attribute | Hib vaccine A | | Hib vaccine B |
| --- | --- | --- | --- |
| Place of origin | Imported product | | Domestic product |
| Effectiveness (%) | 85% | | 95% |
| Adverse events | 5.0/1 million doses (Low) | | 25.0/1 million doses (High) |
| The price of full vaccination | 400 yuan | | 200 yuan |
| Doctor’s recommendation | Recommendation | | No recommendation |
| **Which vaccine would you prefer?** | | | |
| **In reality, would you vaccinate your child with the** **option you chosed above?** | | YES  NO | |

Subject 7

| Attribute | Hib vaccine A | Hib vaccine B |
| --- | --- | --- |
| Place of origin | Imported product | Domestic product |
| Effectiveness (%) | 75% | 95% |
| Adverse events | 25.0/1 million doses (High) | 15.0/1 million doses (Moderate) |
| The price of full vaccination | 800 yuan | 600 yuan |
| Doctor’s recommendation | Recommendation | No recommendation |
| **Which vaccine would you prefer?** | | |
| **In reality, would you vaccinate your child with the** **option you chosed above?** | YES  NO | |

Subject 8

| Attribute | Hib vaccine A | | Hib vaccine B |
| --- | --- | --- | --- |
| Place of origin | Imported product | | Domestic product |
| Effectiveness (%) | 95% | | 85% |
| Adverse events | 5.0/1 million doses (Low) | | 15.0/1 million doses (Moderate) |
| The price of full vaccination | 200 yuan | | 600 yuan |
| Doctor’s recommendation | Recommendation | | No recommendation |
| **Which vaccine would you prefer?** | | | |
| **In reality, would you vaccinate your child with the** **option you chosed above?** | | YES  NO | |

Subject 9

| Attribute | Hib vaccine A | Hib vaccine B |
| --- | --- | --- |
| Place of origin | Imported product | Domestic product |
| Effectiveness (%) | 75% | 95% |
| Adverse events | 15.0/1 million doses (Moderate) | 5.0/1 million doses (Low) |
| The price of full vaccination | 200 yuan | 800 yuan |
| Doctor’s recommendation | No recommendation | Recommendation |
| **Which vaccine would you prefer?** | | |
| **In reality, would you vaccinate your child with the** **option you chosed above?** | YES  NO | |

***Version 2:*** *The questionnaire of discrete choice experiments*

Subject 1

| Attribute | Hib vaccine A | Hib vaccine B |
| --- | --- | --- |
| Place of origin | Imported product | Domestic product |
| Effectiveness (%) | 75% | 95% |
| Adverse events | 5.0/1 million doses (Low) | 25.0/1 million doses (High) |
| The price of full vaccination | 400 yuan | 200 yuan |
| Doctor’s recommendation | No recommendation | Recommendation |
| **Which vaccine would you prefer?** | | |
| **In reality, would you vaccinate your child with the** **option you chosed above?** | YES  NO | |

Subject 2

| Attribute | Hib vaccine A | Hib vaccine B |
| --- | --- | --- |
| Place of origin | Imported product | Domestic product |
| Effectiveness (%) | 75% | 95% |
| Adverse events | 15.0/1 million doses (Moderate) | 25.0/1 million doses (High) |
| The price of full vaccination | 800 yuan | 400 yuan |
| Doctor’s recommendation | No recommendation | Recommendation |
| **Which vaccine would you prefer?** | | |
| **In reality, would you vaccinate your child with the** **option you chosed above?** | YES  NO | |

Subject 3

| Attribute | Hib vaccine A | Hib vaccine B |
| --- | --- | --- |
| Place of origin | Domestic product | Imported product |
| Effectiveness (%) | 85% | 75% |
| Adverse events | 15.0/1 million doses (Moderate) | 25.0/1 million doses (High) |
| The price of full vaccination | 800 yuan | 400 yuan |
| Doctor’s recommendation | Recommendation | No recommendation |
| **Which vaccine would you prefer?** | | |
| **In reality, would you vaccinate your child with the** **option you chosed above?** | YES  NO | |

Subject 4

| Attribute | Hib vaccine A | Hib vaccine B |
| --- | --- | --- |
| Place of origin | Imported product | Domestic product |
| Effectiveness (%) | 95% | 75% |
| Adverse events | 25.0/1 million doses (High) | 5.0/1 million doses (Low) |
| The price of full vaccination | 600 yuan | 200 yuan |
| Doctor’s recommendation | No recommendation | Recommendation |
| **Which vaccine would you prefer?** | | |
| **In reality, would you vaccinate your child with the** **option you chosed above?** | YES  NO | |

Subject 5

| Attribute | Hib vaccine A | Hib vaccine B |
| --- | --- | --- |
| Place of origin | Imported product | Domestic product |
| Effectiveness (%) | 95% | 85% |
| Adverse events | 15.0/1 million doses (Moderate) | 5.0/1 million doses (Low) |
| The price of full vaccination | 800 yuan | 600 yuan |
| Doctor’s recommendation | Recommendation | No recommendation |
| **Which vaccine would you prefer?** | | |
| **In reality, would you vaccinate your child with the** **option you chosed above?** | YES  NO | |

Subject 6

| Attribute | Hib vaccine A | Hib vaccine B |
| --- | --- | --- |
| Place of origin | Imported product | Domestic product |
| Effectiveness (%) | 85% | 95% |
| Adverse events | 25.0/1 million doses (High) | 15.0/1 million doses (Moderate) |
| The price of full vaccination | 800 yuan | 600 yuan |
| Doctor’s recommendation | Recommendation | No recommendation |
| **Which vaccine would you prefer?** | | |
| **In reality, would you vaccinate your child with the** **option you chosed above?** | YES  NO | |

Subject 7

| Attribute | Hib vaccine A | Hib vaccine B |
| --- | --- | --- |
| Place of origin | Domestic product | Imported product |
| Effectiveness (%) | 75% | 85% |
| Adverse events | 5.0/1 million doses (Low) | 25.0/1 million doses (High) |
| The price of full vaccination | 600 yuan | 400 yuan |
| Doctor’s recommendation | No recommendation | Recommendation |
| **Which vaccine would you prefer?** | | |
| **In reality, would you vaccinate your child with the** **option you chosed above?** | YES  NO | |

Subject 8

| Attribute | Hib vaccine A | Hib vaccine B |
| --- | --- | --- |
| Place of origin | Domestic product | Imported product |
| Effectiveness (%) | 85% | 75% |
| Adverse events | 5.0/1 million doses (Low) | 15.0/1 million doses (Moderate) |
| The price of full vaccination | 400 yuan | 600 yuan |
| Doctor’s recommendation | No recommendation | Recommendation |
| **Which vaccine would you prefer?** | | |
| **In reality, would you vaccinate your child with the** **option you chosed above?** | YES  NO | |

Subject 9

| Attribute | Hib vaccine A | Hib vaccine B |
| --- | --- | --- |
| Place of origin | Imported product | Domestic product |
| Effectiveness (%) | 75% | 95% |
| Adverse events | 15.0/1 million doses (Moderate) | 25.0/1 million doses (High) |
| The price of full vaccination | 800 yuan | 400 yuan |
| Doctor’s recommendation | No recommendation | Recommendation |
| **Which vaccine would you prefer?** | | |
| **In reality, would you vaccinate your child with the** **option you chosed above?** | YES  NO | |

***Version 3:*** *The questionnaire of discrete choice experiments*

Subject 1

| Attribute | Hib vaccine A | Hib vaccine B |
| --- | --- | --- |
| Place of origin | Imported product | Domestic product |
| Effectiveness (%) | 75% | 95% |
| Adverse events | 15.0/1 million doses (Moderate) | 25.0/1 million doses (High) |
| The price of full vaccination | 800 yuan | 400 yuan |
| Doctor’s recommendation | No recommendation | Recommendation |
| **Which vaccine would you prefer?** | | |
| **In reality, would you vaccinate your child with the** **option you chosed above?** | YES  NO | |

Subject 2

| Attribute | Hib vaccine A | Hib vaccine B |
| --- | --- | --- |
| Place of origin | Imported product | Domestic product |
| Effectiveness (%) | 75% | 95% |
| Adverse events | 5.0/1 million doses (Low) | 15.0/1 million doses (Moderate) |
| The price of full vaccination | 600 yuan | 400 yuan |
| Doctor’s recommendation | No recommendation | Recommendation |
| **Which vaccine would you prefer?** | | |
| **In reality, would you vaccinate your child with the** **option you chosed above?** | YES  NO | |

Subject 3

| Attribute | Hib vaccine A | Hib vaccine B |
| --- | --- | --- |
| Place of origin | Imported product | Domestic product |
| Effectiveness (%) | 75% | 85% |
| Adverse events | 15.0/1 million doses (Moderate) | 25.0/1 million doses (High) |
| The price of full vaccination | 600 yuan | 200 yuan |
| Doctor’s recommendation | Recommendation | No recommendation |
| **Which vaccine would you prefer?** | | |
| **In reality, would you vaccinate your child with the** **option you chosed above?** | YES  NO | |

Subject 4

| Attribute | Hib vaccine A | Hib vaccine B |
| --- | --- | --- |
| Place of origin | Imported product | Domestic product |
| Effectiveness (%) | 95% | 85% |
| Adverse events | 5.0/1 million doses (Low) | 25.0/1 million doses (High) |
| The price of full vaccination | 400 yuan | 600 yuan |
| Doctor’s recommendation | No recommendation | Recommendation |
| **Which vaccine would you prefer?** | | |
| **In reality, would you vaccinate your child with the** **option you chosed above?** | YES  NO | |

Subject 5

| Attribute | Hib vaccine A | Hib vaccine B |
| --- | --- | --- |
| Place of origin | Imported product | Domestic product |
| Effectiveness (%) | 95% | 75% |
| Adverse events | 25.0/1 million doses (High) | 5.0/1 million doses (Low) |
| The price of full vaccination | 800 yuan | 200 yuan |
| Doctor’s recommendation | No recommendation | Recommendation |
| **Which vaccine would you prefer?** | | |
| **In reality, would you vaccinate your child with the** **option you chosed above?** | YES  NO | |

Subject 6

| Attribute | Hib vaccine A | Hib vaccine B |
| --- | --- | --- |
| Place of origin | Imported product | Domestic product |
| Effectiveness (%) | 75% | 95% |
| Adverse events | 25.0/1 million doses (High) | 5.0/1 million doses (Low) |
| The price of full vaccination | 200 yuan | 400 yuan |
| Doctor’s recommendation | No recommendation | Recommendation |
| **Which vaccine would you prefer?** | | |
| **In reality, would you vaccinate your child with the** **option you chosed above?** | YES  NO | |

Subject 7

| Attribute | Hib vaccine A | Hib vaccine B |
| --- | --- | --- |
| Place of origin | Domestic product | Imported product |
| Effectiveness (%) | 75% | 85% |
| Adverse events | 5.0/1 million doses (Low) | 15.0/1 million doses (Moderate) |
| The price of full vaccination | 200 yuan | 400 yuan |
| Doctor’s recommendation | Recommendation | No recommendation |
| **Which vaccine would you prefer?** | | |
| **In reality, would you vaccinate your child with the** **option you chosed above?** | YES  NO | |

Subject 8

| Attribute | Hib vaccine A | Hib vaccine B |
| --- | --- | --- |
| Place of origin | Domestic product | Imported product |
| Effectiveness (%) | 85% | 95% |
| Adverse events | 15.0/1 million doses (Moderate) | 5.0/1 million doses (Low) |
| The price of full vaccination | 800 yuan | 600 yuan |
| Doctor’s recommendation | No recommendation | Recommendation |
| **Which vaccine would you prefer?** | | |
| **In reality, would you vaccinate your child with the** **option you chosed above?** | YES  NO | |

Subject 9

| Attribute | Hib vaccine A | Hib vaccine B |
| --- | --- | --- |
| Place of origin | Imported product | Domestic product |
| Effectiveness (%) | 75% | 95% |
| Adverse events | 5.0/1 million doses (Low) | 15.0/1 million doses (Moderate) |
| The price of full vaccination | 600 yuan | 400 yuan |
| Doctor’s recommendation | No recommendation | Recommendation |
| **Which vaccine would you prefer?** | | |
| **In reality, would you vaccinate your child with the** **option you chosed above?** | YES  NO | |
